# Assessment of Behavioral Health Services Use Among Low-Income Medicare Beneficiaries After Reductions in Coinsurance Fees

**DOI:** 10.1001/jamanetworkopen.2020.19854

**Published:** 2020-10-08

**Authors:** Vicki Fung, Mary Price, Andrew A. Nierenberg, John Hsu, Joseph P. Newhouse, Benjamin L. Cook

**Affiliations:** 1 The Mongan Institute, Massachusetts General Hospital, Boston; 2 Harvard Medical School, Boston, Massachusetts; 3 Department of Psychiatry, Massachusetts General Hospital, Boston; 4 Department of Health Policy and Management, Harvard T.H. Chan School of Public Health, Boston, Massachusetts; 5 Harvard Kennedy School, Cambridge, Massachusetts; 6 Health Equity Research Lab, Cambridge Health Alliance and Harvard Medical School, Cambridge, Massachusetts

## Abstract

**Importance:**

Medicare has historically imposed higher beneficiary coinsurance for behavioral health services than for medical and surgical care but gradually introduced parity between 2009 and 2014. Although Medicare insures many people with serious mental illness (SMI), there is limited information on the impact of coinsurance parity in this population.

**Objective:**

To examine the association between coinsurance parity and outpatient behavioral health care use among low-income beneficiaries with SMI.

**Design, Setting, and Participants:**

This cohort study used Medicare claims data for a 50% national sample of lower-income Medicare beneficiaries from January 1, 2007, to December 31, 2016. The study sample included patients with SMI (schizophrenia, bipolar disorder, or major depressive disorder). Data analysis was performed from August 1, 2018, to July 15, 2020.

**Exposures:**

Reduction in behavioral health care coinsurance from 50% to 20% between January 1, 2009, and January 1, 2014.

**Main Outcomes and Measures:**

Total annual spending for outpatient behavioral health care visits and the percentage of beneficiaries with an annual outpatient behavioral health care visit overall, with a prescriber, and with a psychiatrist. A difference-in-difference approach was used to compare outcomes before and after the reduction in coinsurance for beneficiaries with and without cost-sharing decreases. Linear regression models with beneficiary fixed effects that adjusted for time-changing beneficiary- and area-level covariates were used to examine changes in outcomes.

**Results:**

The study included 793 275 beneficiaries with SMI in 2008; 518 893 (65.4%) were younger than 65 years (mean [SD] age, 57.6 [16.1] years), 511 265 (64.4%) were female, and 552 056 (69.6%) were White. In 2008, the adjusted percentage of beneficiaries with an outpatient behavioral health care visit was 40.7% (95% CI, 40.4%-41.0%) among those eligible for the cost-sharing reduction and 44.9% (95% CI, 44.9%-45.0%) among those with free care. The mean adjusted out-of-pocket costs for outpatient behavioral health care visits decreased from $132 (95% CI, $129-$136) in 2008 to $64 (95% CI, $61-$66) in 2016 among those with reductions in cost-sharing. The adjusted percentage of beneficiaries with behavioral health care visits increased to 42.2% (95% CI, 41.9%-42.5%) in the group with a reduction in coinsurance and to 47.2% (95% CI, 47.0%-47.3%) in the group with free care. The cost-sharing reduction was not positively associated with visits (eg, relative change of −0.76 percentage points [95% CI, −1.12 to −0.40 percentage points] in the percentage of beneficiaries with outpatient behavioral health care visits in 2016 vs 2008).

**Conclusions and Relevance:**

This cohort study found that beneficiary costs for outpatient behavioral health care decreased between 2009 and 2014. There was no association between cost-sharing reductions and changes in behavioral health care visits. Low levels of use in this high-need population suggest the need for other policy efforts to address additional barriers to behavioral health care.

## Key Points

**Question:**

Was the implementation of behavioral health coinsurance parity in Medicare associated with outpatient behavioral health care use or spending among low-income beneficiaries with serious mental illness?

**Findings:**

In this cohort study of 793 275 Medicare beneficiaries, out-of-pocket costs for outpatient behavioral health care decreased among those with cost-sharing reductions, but behavioral health care use and spending were similar among beneficiaries with reductions in coinsurance for this care and beneficiaries with free care.

**Meaning:**

The findings suggest that additional policies or interventions that target nonfinancial barriers to behavioral health care may be needed to address low levels of use of this care among low-income Medicare beneficiaries with serious mental illness.

## Teaser

This cohort study assesses the association of reductions in coinsurance for behavioral health services with behavioral health care use and spending among Medicare beneficiaries with severe mental illness.

## Introduction

Medicare is a major source of coverage for individuals with disabling mental illness. Many policy efforts to improve access to behavioral health services for both mental health care and substance use treatment have focused on reducing cost-related barriers to care.^1^ The Medicare Improvements for Patients and Providers Act of 2008 required parity in cost-sharing for outpatient behavioral health care. Until 2010, Medicare limited payment for behavioral health services such that beneficiary coinsurance rates were 50% for behavioral health services and 20% for medical and surgical care. Starting in 2010, Medicare reduced behavioral health cost-sharing, to 45% in 2010 and 2011, 40% in 2012, 35% in 2013, and 20% in 2014 onward.^2^

Whether the introduction of cost-sharing parity increased use of behavioral health services for traditional Medicare beneficiaries is not known. One study^3^ found that outpatient follow-up after psychiatric hospitalization was higher among Medicare Advantage beneficiaries enrolled in private plans that voluntarily implemented mental health cost-sharing parity. In contrast, prior parity policies, including in the Mental Health Parity and Addictions Equity Act, and state laws have largely applied to commercial insurance plans and have been associated with greater financial protection for patients but limited increases in behavioral health care use and spending.^4,5,6,7,8^ Some researchers posit that commercial plans increased utilization management approaches to mitigate increases in behavioral health care spending associated with parity.^7^ Traditional fee-for-service Medicare does not impose utilization management restrictions; to the extent that such tactics might have offset use in prior parity policies, there could be larger effects associated with coinsurance reductions in traditional Medicare than in commercial insurance.

A prior analysis^9^ of the Medical Expenditure Panel Survey, however, did not find increases in mental health–related visits for Medicare beneficiaries compared with commercial enrollees associated with Medicare Improvements for Patients and Providers Act but did find increased psychotropic drug use. This analysis was limited to high-income beneficiaries to exclude beneficiaries who might not have been exposed to the parity policy change because of cost-sharing subsidies. Although beneficiaries with income below 100% of the federal poverty level generally qualify for subsidies that cover Medicare cost-sharing, low-income beneficiaries with incomes just above that threshold who have full Medicare cost-sharing and do not have supplementary insurance could be especially affected by changes in out-of-pocket costs for behavioral health care. Moreover, the prevalence of serious mental health and substance use disorders is more common among low-income beneficiaries, and the impact of parity could be greater in higher need populations. We investigated the association of behavioral health care coinsurance parity with cost-sharing levels, outpatient behavioral health care visits, and spending among low-income traditional Medicare beneficiaries with serious mental illness (SMI).

## Methods

### Study Data, Population, and Design

This cohort study used Medicare claims data for a 50% national sample of lower-income Medicare beneficiaries from January 1, 2007, to December 31, 2016. The reduction in behavioral health care coinsurance from 50% to 20% occurred between January 1, 2009, and January 1, 2014. To assess the association of the parity policy with the aforementioned outcomes and control for temporal trends, we compared 2 groups of Medicare beneficiaries: (1) low-income beneficiaries who qualified for full cost-sharing subsidies through the Medicare Savings Program and had no cost-sharing for outpatient behavioral health care services (ie, free care) and (2) beneficiaries with incomes just above the limits for these subsidies who experienced the behavioral health care coinsurance reduction. We compared those just above and below these income thresholds to help reduce potential bias owing to unmeasured differences that could influence outcomes, especially related to socioeconomic status, for beneficiaries with and without the cost-sharing reduction. The Mass General Brigham institutional review board approved the study for secondary use of research samples and data with a waiver of informed consent. This study followed the Strengthening the Reporting of Observational Studies in Epidemiology (STROBE) reporting guideline.

Because we lacked information on beneficiaries’ household incomes, we used data on whether beneficiaries received different income-based subsidies to determine whether beneficiaries were eligible for the behavioral health care cost-sharing reduction or free care. Specifically, those with the cost-sharing reduction included beneficiaries who received premium assistance only (eg, from the Specified Low-Income Medicare Beneficiaries Program) or Medicare Part D full low-income subsidies. The federal income limit for these programs is 135% FPL. The comparison group of those with free care included qualified Medicare beneficiaries, for whom the federal income limit is 100% FPL, and other full-benefit dual-eligible beneficiaries (eTable 1 in the Supplement).

The study cohort included noninstitutionalized, traditional Medicare beneficiaries.^10^ We used a dynamic cohort in which individuals could enter (eg, if they newly received subsidies, entered Medicare, or received an SMI diagnosis) or exit (owing to death, switching to Medicare Advantage, or no longer receiving subsidies) in each year. We required beneficiaries to be enrolled in Medicare with the same subsidy status for the entire year. Data on whether beneficiaries had supplemental coverage that covered Medicare cost-sharing were unavailable, but this is unlikely among low-income beneficiaries.^11^

We focused on beneficiaries with diagnoses of SMI, including schizophrenia, bipolar disorder, or major depressive disorder, using Medicare Chronic Condition Warehouse indicators as of December 31 of the prior year. These algorithms are based on *International Classification of Diseases, Ninth Revision*, *International Statistical Classification of Diseases and Related Health Problems, Tenth Revision*, Medicare Severity Diagnosis Related Groups, and Healthcare Common Procedural Coding System procedure codes and require at least 1 inpatient or 2 outpatient diagnoses over 2 years.^12^ Demographic traits of beneficiaries, including age, sex, reason for entitlement, and race/ethnicity, were obtained from the Master Beneficiary Summary File.^13^

### Outcomes

We identified outpatient visits using place of service in professional claims (eg, office) and bill type in outpatient facility claims (eg, hospital outpatient departments, federally qualified health centers, or rural health centers). To identify behavioral health visits, we included visits with *International Classification of Diseases, Ninth Revision, Clinical Modification* codes of 290-319 and corresponding *International Statistical Classification of Diseases, Tenth Revision, Clinical Modification* codes before and after October 2015.

For behavioral health care visits, we calculated 2 spending measures: (1) total annual spending, using the Medicare allowed amount, which includes both Medicare and beneficiary costs, and (2) beneficiary out-of-pocket costs, which we calculated as the allowed amount less the Medicare-covered amount. For visits including multiple services, the mental health reimbursement limitation only applied to the services associated with behavioral health diagnoses. For beneficiaries with full cost-sharing subsidies, we assumed out-of-pocket costs were 0. Costs are adjusted to 2016 dollars using the all-items Consumer Price Index.

We examined behavioral health care visits with any health care professional in each year and with prescribers and psychiatrists because medications represent a cornerstone of treatment for many beneficiaries with SMI. Prescribers included psychiatrists, primary care physicians, nurse practitioners, and visits to outpatient facilities; provider specialty information is not in facility claims; thus, we may have overestimated prescriber visits.

Over the study period, there were large fluctuations in claims for community mental health centers (CMHCs) associated with detected fraud and subsequent monitoring.^14^ This monitoring was associated with reductions in CMHC billing to Medicare from $273 million in 2008 to $31 million in 2012, with larger reductions among those with free care than among those with cost-sharing reductions (eFigure 1 in the Supplement). Because these changes in CMHC visits were unlikely to be associated with the parity policy change, we excluded these visits from the analysis to mitigate potential bias from this unrelated change. However, we may have undercounted visit rates because we could not distinguish fraudulent from nonfraudulent activity, especially in the earlier years of the study; however, these visits accounted for a small proportion of visits (eg, <7.0% of all behavioral health visits in 2016) (eFigure 1 in the Supplement).

### Statistical Analysis

Data analysis was performed from August 1, 2018, to July 15, 2020. We estimated changes in the likelihood that beneficiaries had at least 1 behavioral health visit per year, which represents a lower bound of care needed to manage SMI diagnoses even if beneficiaries have reached a steady state in their therapeutic management (eg, annual mediation review). In secondary analyses, we examined the number of behavioral health visit per beneficiary per year. We used a difference-in-difference approach to assess changes in years after parity implementation vs preimplementation for those exposed vs those unexposed. Because the policy was phased in over time, we estimated changes in outcomes in each year from 2009 to 2016 vs 2008 using linear regression models with beneficiary-level fixed effects to account for both measured and unmeasured time-stable differences between the groups. The models adjusted for parity exposure status, year indicators, and exposure × year interactions (main coefficient of interest). We allowed parity exposure status to change annually; 8.1% of the sample switched between the cost-sharing reduction and free care groups during the study period.

The models also adjusted for potential time-changing confounders, updated annually, including Centers for Medicare & Medicaid Services Hierarchical Condition Category comorbidity scores, SMI diagnosis indicators, time since SMI diagnosis, whether the beneficiary was assigned to an accountable care organization, whether the beneficiary lived in a rural area (using the US Department of Agriculture rural-urban commuting area codes), whether they lived in a low socioeconomic status ZIP code (ie, >20% of households below the poverty level or >25% of adults ≥25 years of age with a high school education or less using American Community Survey data), state of residence, and state × year interactions.

The state × year fixed effects allowed us to estimate changes over time for beneficiaries with and without the cost-sharing reduction living in the same state and to reduce potential confounding associated with other concurrent policy changes, such as Medicaid expansion. The magnitude of coverage expansion associated with the Patient Protection and Affordable Care Act differed substantially across states, especially owing to differences in Medicaid expansion decisions, which could be associated with differential crowd-out of care (eg, reduced availability of behavioral health physicians for Medicare beneficiaries owing to increased demand for care from individuals newly gaining coverage) across states. In addition, comparison groups that were limited to low-income beneficiaries were included to help address potential confounding owing to crowd-out because they are more likely to live in similar neighborhoods and use similar physicians. Analyses were performed using Stata, version 16.1 (StataCorp, LLC).

To examine potential differences by diagnosis, we conducted sensitivity analyses limited to those with SMI diagnoses of major depressive disorder only. Payments to health care professionals who treated beneficiaries with free care (control group) may have increased with parity implementation in some states (eMethods in the Supplement). To address potential changes in use in the control group associated with these changes, we conducted sensitivity analyses focusing on beneficiaries living in states where payments were less likely to change owing to state reimbursement policies.^15^

## Results

Our sample included 793 275 beneficiaries in the baseline year, 2008 (Table 1). A total of 518 893 (65.4%; 92 060 [60.1%] of those with the cost-sharing reduction and 426 833 [66.7%] of those with free care) were younger than age 65 years (mean [SD] age, 57.6 [16.1] years), 511 265 (64.4%; 97 116 [63.4%] with the reduction and 414 149 [64.7%] with free care) were female, and 552 056 (69.6%; 118 666 [77.5%] with the reduction and 433 390 [67.7%] with free care) were White. A total of 109 660 beneficiaries in the cost-sharing reduction group (71.6%) and 486 386 beneficiaries in the free care group originally qualified (76.0%) for Medicare owing to disability. Compared with beneficiaries with free care, those with the cost-sharing reduction were less likely to have diagnoses of schizophrenia (24 893 [16.3%] vs 154 457 [24.1%]) and bipolar disorder (32 887 [21.5%] vs 169 957 [26.5%]) and had lower mean comorbidity scores (1.18 vs 1.32). In 2016, 52 190 beneficiaries in the cost-sharing reduction group (22.4%) and 170 183 in the free care group (20.6%) were assigned to an accountable care organization.

**Table 1.** Study Population Characteristics in 2008^a^

Characteristic	Cost-sharing reduction (n = 153 070)	Free care (n = 640 205)
Age group, y		
<65	92 060 (60.1)	426 833 (66.7)
65-74	29 359 (19.2)	104 717 (16.4)
75-84	22 870 (14.9)	78 322 (12.2)
≥85	8781 (5.7)	30 333 (4.7)
Female	97 116 (63.4)	414 149 (64.7)
Original reason for entitlement		
Age	41 764 (27.3)	145 609 (22.7)
Disability	109 660 (71.6)	486 386 (76.0)
ESKD and disability or ESKD only	1646 (1.1)	8210 (1.3)
Race/ethnicity		
White	118 666 (77.5)	433 390 (67.7)
Black	21 439 (14.0)	99 861 (15.6)
Hispanic	9476 (6.2)	77 208 (12.1)
Asian or Pacific Islander	1105 (0.7)	16 722 (2.6)
Other	2384 (1.6)	13 024 (2.0)
Diagnosis		
Schizophrenia	24 893 (16.3)	154 457 (24.1)
Bipolar disorder	32 887 (21.5)	169 957 (26.5)
Major depressive disorder	132 643 (86.7)	535 192 (83.6)
ZIP code trait		
Rural^b^	47 103 (30.8)	167 018 (26.1)
Low SES^c^	13 768 (9.0)	73 971 (11.6)
HCC comorbidity score, mean (SD)	1.18 (0.95)	1.32 (1.05)
Years since first SMI diagnosis, mean (SD)	4.88 (2.78)	5.28 (2.84)

Abbreviations: ESKD, end-stage kidney disease; HCC, Hierarchical Condition Category; SES, socioeconomic status; SMI, serious mental illness.

^a^ Data are presented as number (percentage) of beneficiaries unless otherwise indicated. The cost-sharing reduction group included beneficiaries with premium and/or Part D subsidies but not cost-sharing subsidies (federal income limit of 135% of the federal poverty level). The free care group included those receiving full cost-sharing subsidies (federal income limit of 100% of the federal poverty level).

^b^ ZIP codes were classified as rural if they contained census tracts with rural-urban commuting area codes greater than 3.

^c^ A total of 4855 and 27 298 partial and full subsidy beneficiaries, respectively, had missing ZIP code data on SES.

### Annual Outpatient Behavioral Health Care Spending

**Figure 1.**
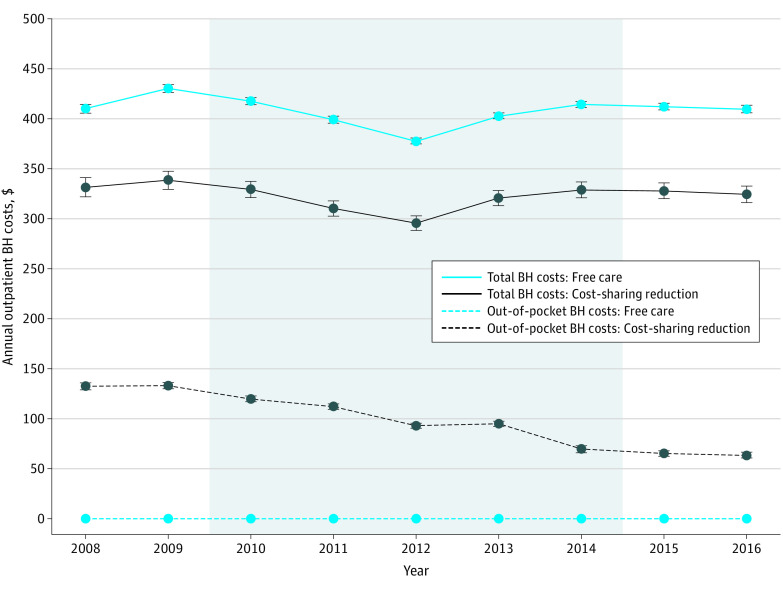
Adjusted Annual Outpatient Behavioral Health (BH) Care Visit Costs Among Beneficiaries With the Cost-Sharing Reduction vs Free Care From 2008 to 2016 Shaded area represents the parity implementation period. All costs are in 2016 US dollars. Error bars indicate 95% CIs.

Figure 1 presents the adjusted annual behavioral health care spending from 2008 to 2016. Total spending in both groups was highest in 2009, the year before parity implementation ($338 [95% CI, $330-$347] and $430 [95% CI, $426-$434] among those with the cost-sharing reductions and free care, respectively); spending was lowest for both groups in 2012. For beneficiaries with the cost-sharing reduction, mean adjusted annual patient out-of-pocket costs for outpatient visits for behavioral health diagnoses were similar in the 2 preparity years and then decreased consistently during the parity implementation period from $133 (95% CI, $130-$136) in 2009 to $65 (95% CI, $62-$68) in 2015, representing a decrease in the proportion of costs paid by beneficiaries from 39.3% to 19.8%.

### Annual Outpatient Behavioral Health Care Visits

**Figure 2.**
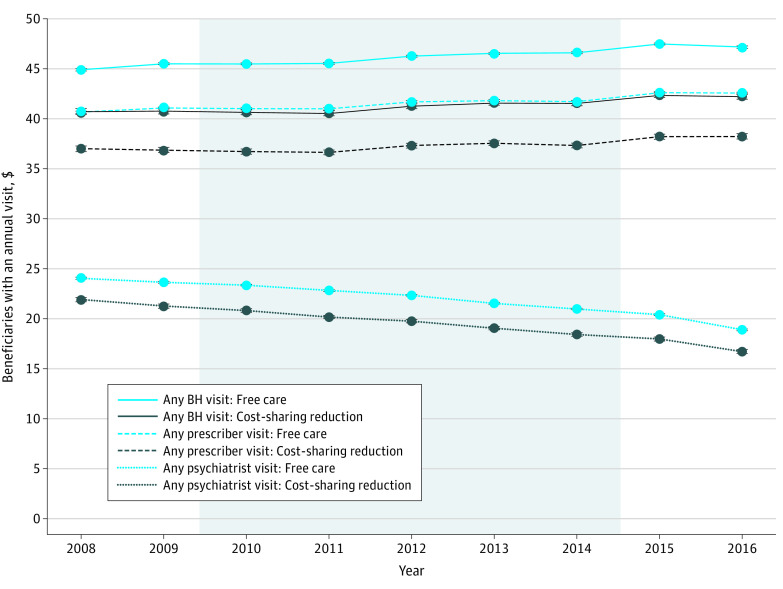
Adjusted Percentage of Beneficiaries With Annual Behavioral Health (BH) Care Visits Among Beneficiaries With the Cost-Sharing Reduction vs Free Care Shaded area represents the parity implementation period. Error bars indicate 95% CIs.

After adjustment, 40.7% (95% CI, 40.4%-41.0%) and 44.9% (95% CI, 44.8%-45.0%) of beneficiaries with the cost-sharing reduction and free care, respectively, had any outpatient behavioral health care visits in 2008 (Figure 2). This percentage increased slightly over time in both groups; in 2016, 42.2% (95% CI, 41.9%-42.5%) in the cost-sharing reduction group and 47.2% (95% CI, 47.0%-47.3%) in the free care group had any visit (44.8% [95% CI, 44.6%-45.1%] and 50.0% [95% CI, 49.9%-50.1%], respectively, if CMHC visits were included) (eFigure 1 in the Supplement). The adjusted mean outpatient behavioral health visits per beneficiary per year decreased slightly from 2.7 (95% CI, 2.6-2.7) to 2.4 (95% CI, 2.4-2.5) for those with the cost-sharing reduction and 3.3 (95% CI, 3.3-3.3) to 3.1 (95% CI, 3.1-3.1) for those with free care between 2008 and 2016 (eFigure 2 in the Supplement). In 2008, 37.0% (95% CI, 36.7%-37.3%) of beneficiaries in the cost-sharing reduction group and 40.6% (95% CI, 40.5%-40.8%) in the free care group had 1 or more visit with a prescriber, which followed a trend similar to that for all behavioral health care visits. The proportion of beneficiaries with 1 or more annual visit with a psychiatrist, however, decreased over time from 21.9% (95% CI, 21.7%-22.1%) in 2008 to 16.7% (95% CI, 16.5%-16.9%) in 2016 among those with the cost-sharing reduction and from 24.0% (95% CI, 23.9%-24.1%) in 2008 to 18.9% (95% CI, 18.8%-19.0%) in 2018 among those with free care.

### Associations Between Behavioral Health Care Parity and Outcomes

In 2009, before parity implementation, relative spending among those with the cost-sharing reduction compared with those with free care decreased by $13 (95% CI, –$20 to –$6) vs 2008 despite no change in coinsurance levels in that year (Table 2). There were also relative reductions in outpatient behavioral health care spending among those with the cost-sharing reduction compared with those with free care in the first 2 parity implementation years when coinsurance decreased from 50% to 45% (difference-in-difference in 2010 vs 2008, $–10; 95% CI, –$19 to –$1). In subsequent years of parity implementation (2012-2014) when coinsurance was further reduced to 40%, 35%, and 20%, and in the years after parity implementation (2015-2016), there were no significant changes in spending.

**Table 2.** Relative Changes in Outpatient BH Care Spending and Annual BH Care Visits Among Beneficiaries With the Cost-Sharing Reduction vs Those With Free Care, 2009-2016 vs 2008

Period	Difference-in-Difference **(**95% CI**)**			
	Total annual BH outpatient spending, $	Any annual BH visit, percentage points	Any annual prescriber visit, percentage points	Any annual psychiatrist visit, percentage points
Preparity				
2009	–13 (–20 to –6)	–0.56 (–0.84 to –0.29)	–0.53 (–0.80 to –0.26)	–0.25 (–0.43 to –0.07)
Parity phase-in				
2010	–10 (–19 to –1)	–0.65 (–0.95 to –0.35)	–0.61 (–0.90 to –0.32)	–0.40 (–0.60 to –0.19)
2011	–10 (–20 to –0.40)	–0.80 (–1.12 to –0.49)	–0.68 (–0.98 to –0.37)	–0.51 (–0.73 to –0.28)
2012	–3 (–13 to 7)	–0.81 (–1.13 to –0.49)	–0.72 (–1.04 to –0.41)	–0.44 (–0.68 to –0.20)
2013	–3 (–14 to 8)	–0.73 (–1.07 to –0.40)	–0.60 (–0.92 to –0.27)	–0.31 (–0.55 to –0.06)
2014	–7 (–18 to 4)	–0.87 (–1.21 to –0.53)	–0.75 (–1.08 to –0.41)	–0.41 (–0.66 to –0.15)
Postparity				
2015	–6 (–17 to 6)	–0.91 (–1.27 to –0.56)	–0.74 (–1.08 to –0.39)	–0.31 (–0.58 to –0.05)
2016	–7 (–18 to 5)	–0.76 (–1.12 to –0.40)	–0.64 (–1.00 to –0.29)	–0.04 (–0.31 to 0.24)

Abbreviation: BH, behavioral health.

Similarly, the relative percentage of beneficiaries who had 1 or more annual outpatient behavioral health care visit in each year decreased by less than 1 percentage point among those with the cost-sharing reduction compared with those with free care in each year vs 2008 (Table 2). These findings were similar for visits with prescribers and psychiatrists and in secondary analyses that examined changes in the number of behavioral health care visits per beneficiary per year (eFigure 3 in the Supplement). Findings were also similar in sensitivity analyses among beneficiaries with major depressive disorder only (eFigure 4 and eTable 2 in the Supplement) and among those living in states in which health care professional payments for the control group were less likely to change with parity implementation (eFigure 5 in the Supplement).

## Discussion

Starting in 2010, Medicare phased out higher coinsurance for outpatient behavioral health care than for medical and surgical services over 5 years. Parity was associated with reduced annual patient out-of-pocket costs for outpatient behavioral health care, by about half among lower-income beneficiaries with SMI, which could have important implications for the affordability of care. However, the policy was not associated with increased relative behavioral health service use or spending among those who experienced the coinsurance reduction compared with a concurrent control group who had no changes in cost-sharing.

Our findings add to the small and mixed body of evidence on the consequences of parity for patients with SMI. For example, in a prior study^7^ among people with bipolar disorder and major depression, utilization did not change in association with parity implementation. The authors attributed this lack of association to potential increases in utilization management approaches by managed care plans. In a different study^16^ of a parity law in Oregon for commercial enrollees, where such managed care practices were less likely, behavioral health care expenditures increased among people with SMI but not in the general population. In the sample of low-income Medicare beneficiaries with SMI in the present study, we found no increase in behavioral health use or spending associated with parity implementation, even within a fee-for-service context with little utilization management. These findings may indicate that the 20% coinsurance continues to present a financial barrier to care for this low-income population or that, with reduced out-of-pocket costs, beneficiaries shifted their spending elsewhere. In a separate analysis, we also did not find an association between parity and visit rates among high-income beneficiaries, suggesting that other, nonfinancial barriers to behavioral health care could continue to limit use.^9^ This could be especially true for patients with SMI for whom innovative, community-based strategies may be needed to increase connections with care.

Despite having comprehensive insurance coverage, outpatient behavioral health care use among beneficiaries with SMI was low throughout the study period, including for those with free care. At baseline, about half or fewer had at least 1 annual outpatient behavioral health care visit with any health care professional type, including psychiatrists, primary care physicians, psychologists, nurse practitioners, and social workers; fewer than one-quarter had an annual visit with a psychiatrist. In contrast, in a prior study^7^ of commercially insured enrollees with bipolar disorder and major depression, more than 80% used behavioral health services. Commonly cited nonfinancial barriers to behavioral health care may be greater in this disadvantaged population, including greater difficulty accessing specialty behavioral health care providers owing to local supply constraints, lack of insurance participation, or fragmented care delivery,^17^ and attitudinal or knowledge barriers, such as lack of perceived need for treatment, stigma concerns, or distrust of physicians.^18^

The national shortage of specialty behavioral health care professionals is well documented, and an aging psychiatry workforce could exacerbate the situation over time.^19,20,21^ Our findings, coupled with the scarcity of behavioral health care professionals in the US, particularly in rural and low-income areas, and concerns about low reimbursement rates for mental health care professionals suggest that reducing behavioral health care cost-sharing may not be sufficient to address access barriers if there is an insufficient health care professional supply to meet increasing demand for services, especially for low-income populations.^22^ In the present study, visits with psychiatrists decreased over time in both groups, and visits with prescribers increased slightly, suggesting that primary care physicians, nurse practitioners, and facilities, such as community clinics, are increasingly providing behavioral health care to this low-income population with SMI. Policies that target increasing the availability of such health care professionals for behavioral health care could be especially effective, such as by expanding access to and reimbursement for telehealth services or virtual consultations and reducing restrictions on the scope of practice for nonphysician health care professionals.^23^

### Limitations

This study has limitations. This was a nonrandomized study, and there could be residual confounding. There were numerous policy changes over the study period, including Patient Protection and Affordable Care Act coverage expansion, that could have affected the availability of behavioral health care professionals for Medicare beneficiaries. Unrelated changes in care from CMHCs owing to fraud enforcement also occurred during this period. To address these concerns, we focused on low-income beneficiaries who could seek care from similar health care professionals and compared beneficiaries living in the same state to account for differences in local coverage expansion or care patterns. However, our analyses did not account for detailed, local measures of behavioral health care professional supply, which could moderate the associations between cost-sharing reductions and use. In addition, we used claims data, which have limited clinical information (eg, to distinguish disease severity or receipt of guideline-concordant care) and limited information on social determinants of health. There could also be undercoding of behavioral health diagnoses, especially in primary care visits for beneficiaries presenting with multiple comorbidities, and the out-of-pocket costs reflect patient liability, not actual paid amounts.

## Conclusions

In this cohort study, the implementation of coinsurance parity was not associated with increased use of behavioral health care services among low-income Medicare beneficiaries with SMI. Although the policy change improved the affordability of care, behavioral health care visit rates remained low, suggesting that other policy efforts are needed to improve outpatient linkages for high-need, disadvantaged Medicare beneficiaries.

## Notes

Supplement. **eTable 1.** Eligibility criteria for Medicare subsidies and Medicaid benefits **eFigure 1.** Adjusted percent of beneficiaries with annual BH visits for beneficiaries with the cost-sharing reduction vs. free care, with and without including CMHC visits **eMethods.** Description of potential changes in provider payments associated with parity change for control group of beneficiaries with free care **eFigure 2.** Adjusted mean annual outpatient BH visits for beneficiaries with the cost-sharing reduction vs. free care, 2008-2016 **eFigure 3.** Diff-in-diff estimates: Relative changes in mean annual BH visits for beneficiaries with the cost-sharing reduction vs. free care 2009-2016 vs. 2008 **eFigure 4.** Sensitivity analysis: Adjusted annual outpatient behavioral health visit cost and percent of beneficiaries with annual BH visits for beneficiaries with the cost-sharing reduction vs. free care, among beneficiaries with major depressive disorder only **eTable 2.** Sensitivity analysis: Relative changes in outpatient BH spending and annual BH visits for beneficiaries with the cost-sharing reduction vs. free care, 2009-2016 vs. 2008 among beneficiaries with major depressive disorder only **eFigure 5.** Sensitivity analysis: Comparison of model results in all states vs. subset of states without payment changes for beneficiaries with full cost-sharing subsidies
