# Supplementary material for: Assessment of Behavioral Health Services Use Among Low-Income Medicare Beneficiaries After Reductions in Coinsurance Fees
Source: JAMA Netw Open. 2020 Oct 8;3(10):e2019854. doi: 10.1001/jamanetworkopen.2020.19854 (PMC7545309; doi:10.1001/jamanetworkopen.2020.19854)
Supplement: Supplement. — eTable 1. Eligibility criteria for Medicare subsidies and Medicaid benefits eFigure 1. Adjusted percent of beneficiaries with annual BH visits for beneficiaries with the cost-sharing reduction vs. free care, with and without including CMHC visits eMethods. Description of potential changes in provider payments associated with parity change for control group of beneficiaries with free care eFigure 2. Adjusted mean annual outpatient BH visits for beneficiaries with the cost-sharing reduction vs. free care, 2008-2016 eFigure 3. Diff-in-diff estimates: Relative changes in mean annual BH visits for beneficiaries with the cost-sharing reduction vs. free care 2009-2016 vs. 2008 eFigure 4. Sensitivity analysis: Adjusted annual outpatient behavioral health visit cost and percent of beneficiaries with annual BH visits for beneficiaries with the cost-sharing reduction vs. free care, among beneficiaries with major depressive disorder only eTable 2. Sensitivity analysis: Relative changes in outpatient BH spending and annual BH visits for beneficiaries with the cost-sharing reduction vs. free care, 2009-2016 vs. 2008 among beneficiaries with major depressive disorder only eFigure 5. Sensitivity analysis: Comparison of model results in all states vs. subset of states without payment changes for beneficiaries with full cost-sharing subsidies [file jamanetwopen-e2019854-s001.pdf]

Supplemental Online Content

Fung V, Price M, Nierenberg AA, Hsu J, Newhouse JP, Cook BL. Assessment of behavioral health services use among low-income Medicare beneficiaries after reductions in coinsurance fees. *JAMA Netw Open*. 2020;3(10):e2019854. doi:10.1001/jamanetworkopen.2020.19854

eTable 1. Eligibility criteria for Medicare subsidies and Medicaid benefits
eFigure 1. Adjusted percent of beneficiaries with annual BH visits for beneficiaries with the cost-sharing reduction vs. free care, with and without including CMHC visits
eMethods. Description of potential changes in provider payments associated with parity change for control group of beneficiaries with free care
eFigure 2. Adjusted mean annual outpatient BH visits for beneficiaries with the cost-sharing reduction vs. free care, 2008-2016
eFigure 3. Diff-in-diff estimates: Relative changes in mean annual BH visits for beneficiaries with the cost-sharing reduction vs. free care 2009-2016 vs. 2008
eFigure 4. Sensitivity analysis: Adjusted annual outpatient behavioral health visit cost and percent of beneficiaries with annual BH visits for beneficiaries with the cost-sharing reduction vs. free care, among beneficiaries with major depressive disorder only
eTable 2. Sensistivity analysis: Relative changes in outpatient BH spending and annual BH visits for beneficiaries with the cost-sharing reduction vs. free care, 2009-2016 vs. 2008 among beneficiaries with major depressive disorder only
eFigure 5. Sensitivity analysis: Comparison of model results in all states vs. subset of states without payment changes for beneficiaries with full cost-sharing subsidies

This supplemental material has been provided by the authors to give readers additional information about their work.

© 2020 Fung V et al. *JAMA Network Open.*

eTable 1. Eligibility criteria for Medicare subsidies and Medicaid benefits

Study Group	Low income subsidy	Income[b]	Medicaid benefits	Part B subsidy	Exposed to BH parity cost-sharing change	Part D subsidy (2014 copays)
Free care: Full cost-sharing subsidy (control group)	QMB Plus; QMB Only; SLMB Plus; Other FBDE[a,c]	≤100% FPL	Yes, except QMB only[a]	Premium + cost-sharing	**No**	Premium + ≤$2.55/ ≤$6.35 copay
Cost-sharing reduction: Partial subsidies (parity exposed group)	SLMB Only; QI; Part D LIS[a,d]	101-135% FPL	No	Premium only or None	**Yes**	Premium + ≤$2.55/ ≤$6.35 copay
No Subsidy (excluded from study)	None	≥135% FPL	No	None	**Yes**	None

[a] QMB=Qualified Medicare Beneficiary; SLMB=Specified Low Income Medicare Beneficiary; QI=Qualifying Individual; LIS=Low income subsidy
[b] Federal limits
[c] Eligibility for other Full Benefit Dual Eligibles (FBDE) varies by state.
[d] A small % receive Partial Part D LIS (135-150% FPL); we will include in sensitivity analyses.

© 2020 Fung V et al. *JAMA Network Open.*

eFigure 1. Adjusted percent of beneficiaries with annual BH visits for beneficiaries with the cost-sharing reduction vs. free care, with and without including CMHC visits [a]

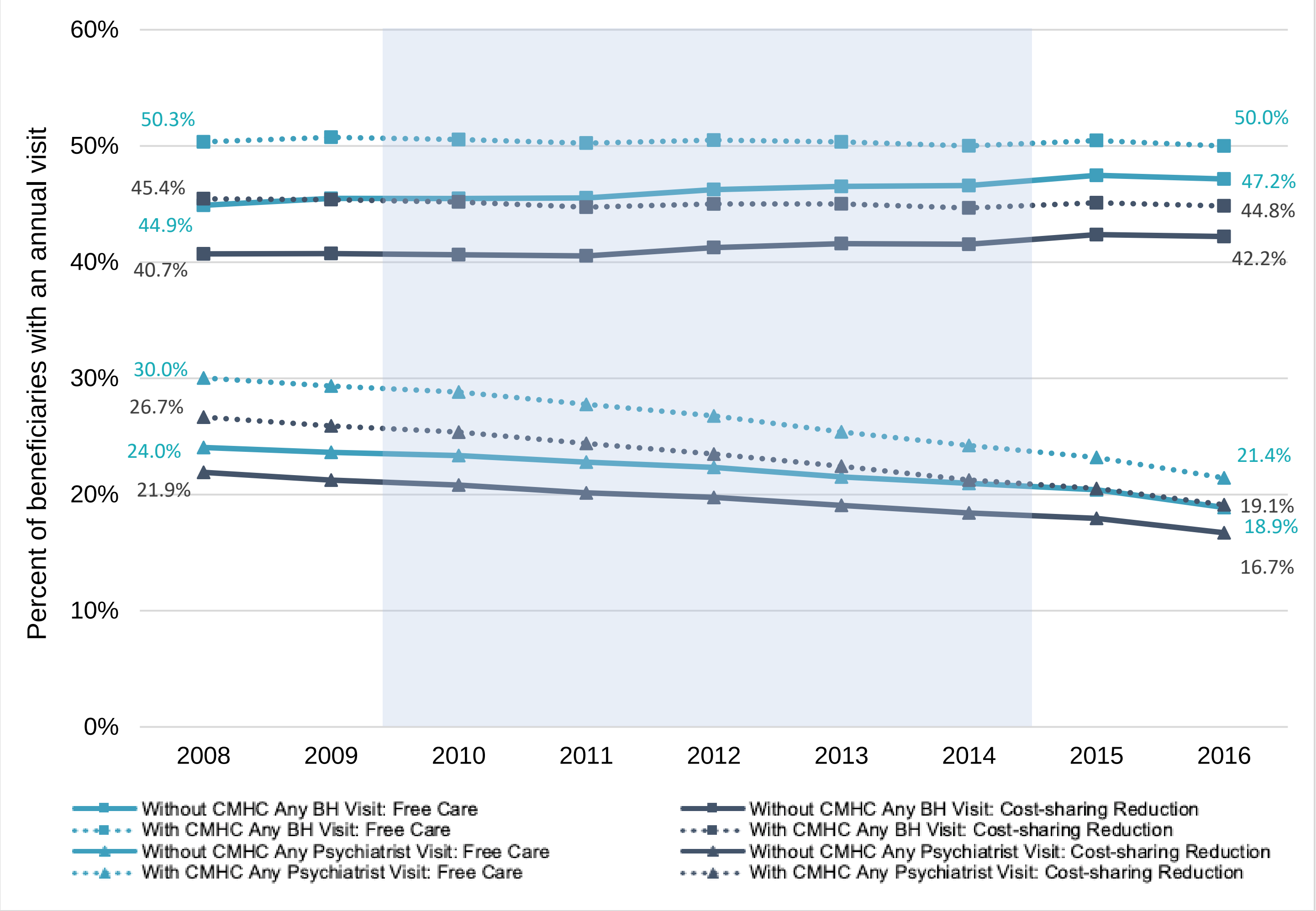

[a] Shaded area represents the parity implementation period.

© 2020 Fung V et al. *JAMA Network Open.*

eMethods. Description of potential changes in provider payments associated with parity change for control group of beneficiaries with free care

For beneficiaries with free care (control group), state Medicaid programs provide wrap around coverage to cover the cost of Medicare cost-sharing. However, many state Medicaid programs pay only the lesser of the Medicare or Medicaid fees for a service. Data on states' Medicaid-Medicare fee ratios for BH services are not readily available; however, the average fee ratio for primary care services in 2016 was 0.66.[1] Thus, if in a hypothetical example where the Medicare fee for a BH visit was $100, in the pre-parity period, Medicare would pay $50 and the state would cover $16 (using the average fee ratio of 0.66). The remaining $34 would be unpaid. In the post-parity period, Medicare covers 80% or $80 of the fee; in our example, the state would now pay nothing, and $20 would be unpaid. There is wide variation in states' Medicaid fees across services, which would impact the timing and magnitude of potential payment increases when caring for QMBs and full benefit duals.

© 2020 Fung V et al. *JAMA Network Open.*

eFigure 2. Adjusted mean annual outpatient BH visits for beneficiaries with the cost-sharing reduction vs. free care, 2008-2016 [a]

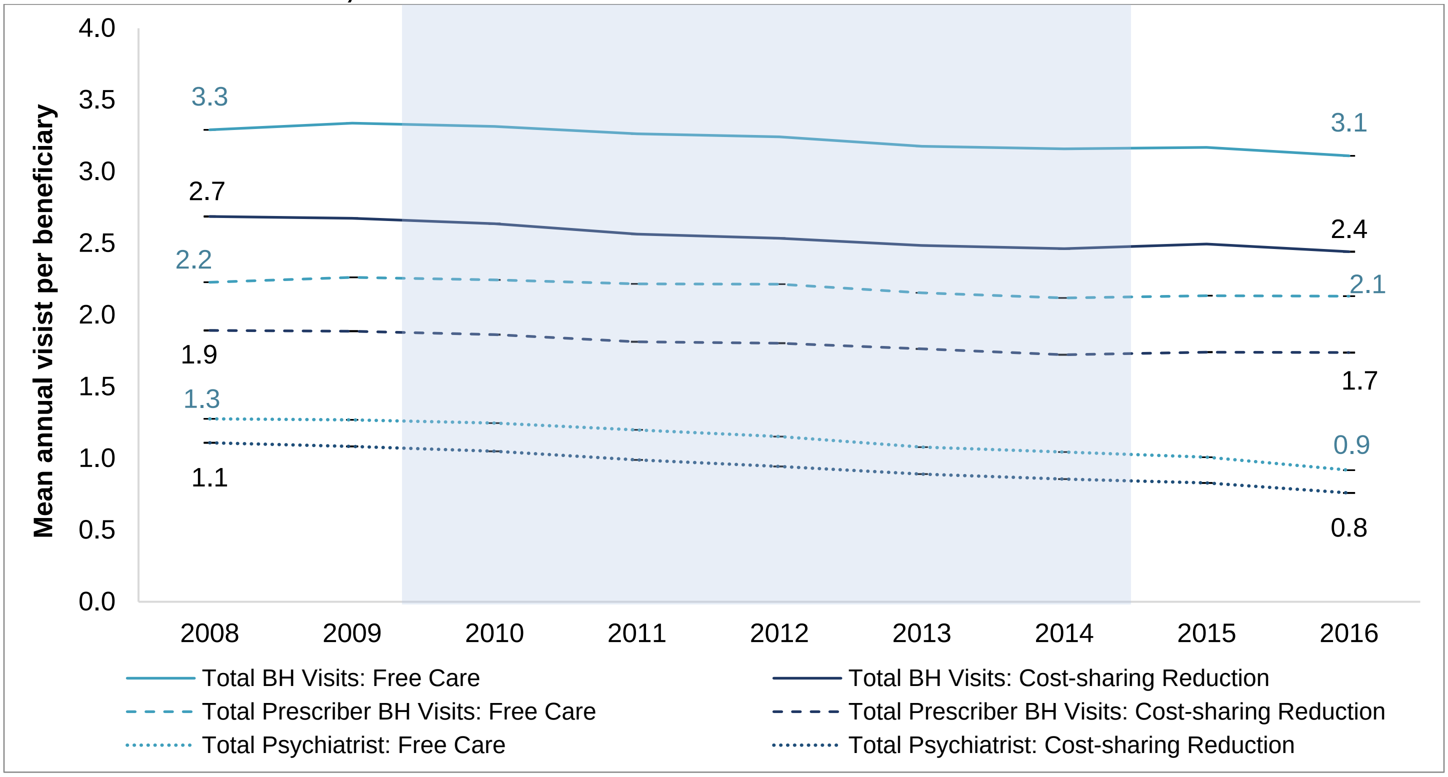

[a] Shaded area represents the parity implementation period.

© 2020 Fung V et al. *JAMA Network Open.*

eFigure 3. Diff-in-Diff estimates: Relative changes in mean annual BH visits for beneficiaries with the cost-sharing reduction vs. free care 2009-2016 vs. 2008

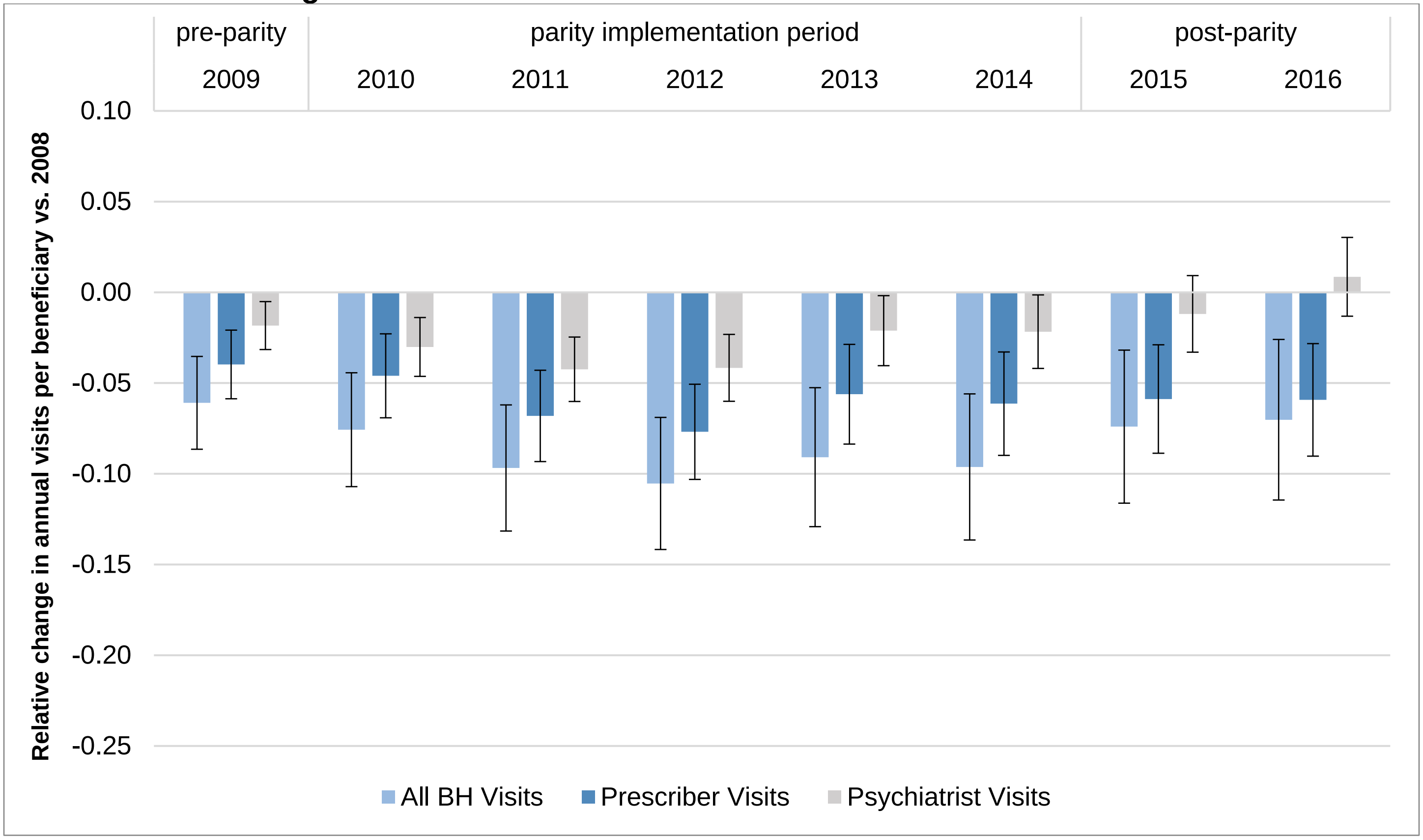

© 2020 Fung V et al. *JAMA Network Open.*

eFigure 4. Sensitivity analysis: Adjusted annual outpatient behavioral health visit cost and percent of beneficiaries with annual BH visits for beneficiaries with the cost-sharing reduction vs. free care, among beneficiaries with major depressive disorder only [a,b]

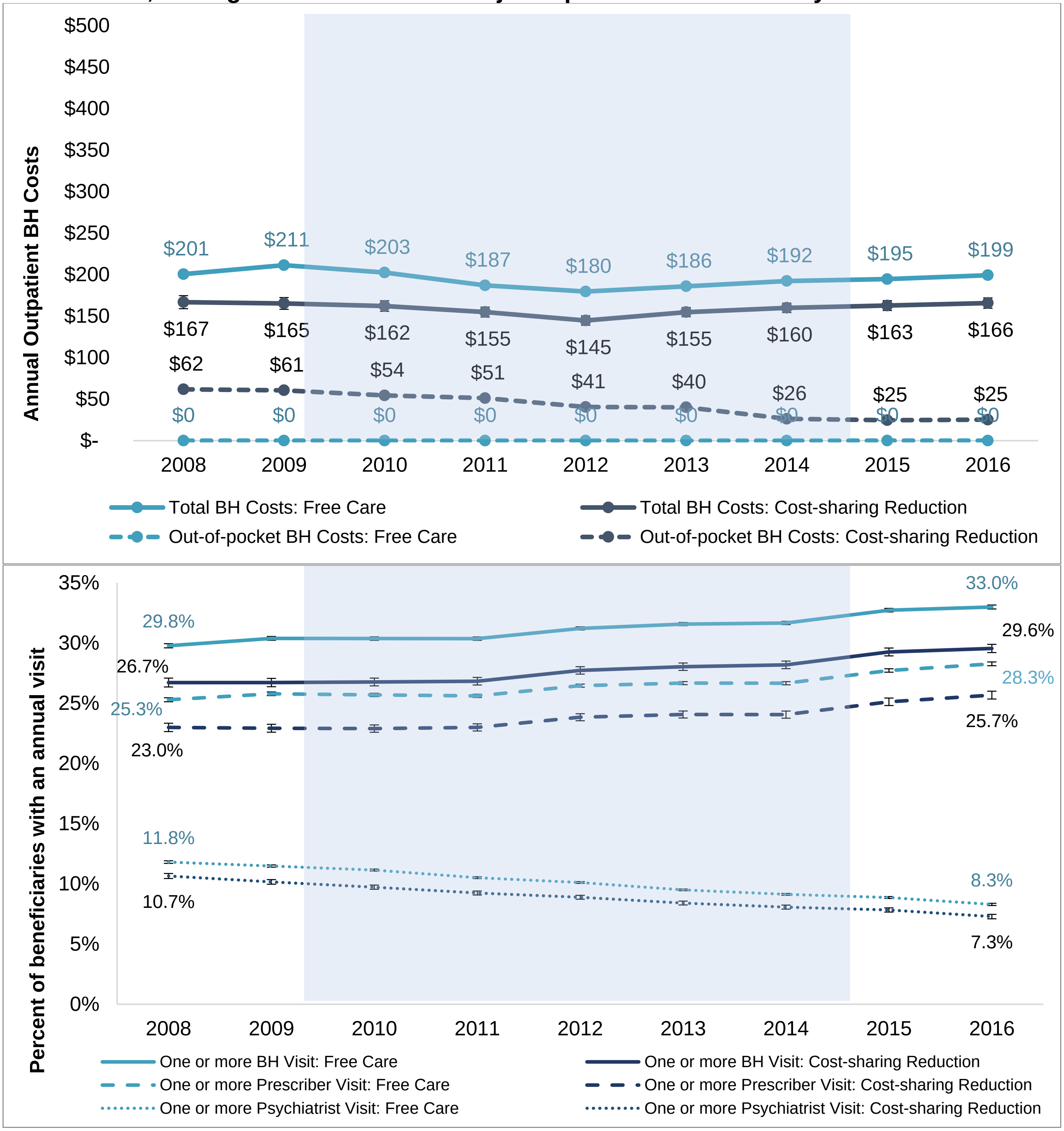

[a] Shaded area represents the parity implementation period.
[b] Bars indicated 95% confidence intervals (not all are visible).

© 2020 Fung V et al. *JAMA Network Open.*

eTable 2. Sensitivity analysis: Relative changes in outpatient BH spending and annual BH visits for beneficiaries with the cost-sharing reduction vs. free care, 2009-2016 vs. 2008 among beneficiaries with major depressive disorder only

	**Total Annual BH Outpatient Spending**			**Percentage with Any Annual BH Visit**			**Percentage with Any Annual Prescriber Visit**			**Percentage with Any Annual Psychiatrist Visit**		
**Year vs. 2008:**	Diff-in-Diff ($)	95%	CI	Diff-in-Diff (pp[a])	95%	CI	Diff-in-Diff (pp[a])	95%	CI	Diff-in-Diff (pp[a])	95%	CI
**Pre-parity**												
2009	**-$12**	**-$19**	**-$6**	**-0.61**	**-0.96**	**-0.27**	**-0.57**	**-0.89**	**-0.24**	-0.16	-0.33	0.01
**Parity phase-in**												
2010	-$7	-$14	$1	**-0.55**	**-0.93**	**-0.17**	**-0.50**	**-0.85**	**-0.14**	**-0.26**	**-0.46**	**-0.07**
2011	$2	-$7	$10	**-0.48**	**-0.88**	**-0.08**	-0.32	-0.70	0.05	-0.11	-0.33	0.10
2012	-$1	-$10	$8	**-0.43**	**-0.84**	**-0.03**	-0.33	-0.71	0.06	-0.07	-0.29	0.16
2013	$3	-$6	$12	**-0.49**	**-0.91**	**-0.06**	-0.30	-0.70	0.09	0.07	-0.17	0.30
2014	$1	-$8	$11	-0.42	-0.85	0.01	-0.31	-0.72	0.10	0.10	-0.14	0.34
**Post-parity**												
2015	$2	-$8	$11	-0.41	-0.86	0.03	-0.30	-0.72	0.12	0.13	-0.12	0.38
2016	$0	-$9	$10	-0.39	-0.85	0.07	-0.30	-0.73	0.14	0.15	-0.11	0.40

[a] pp = percentage point

© 2020 Fung V et al. *JAMA Network Open.*

eFigure 5. Sensitivity analysis: Comparison of model results in all states vs. subset of states without payment changes for beneficiaries with full cost-sharing subsidies[a]

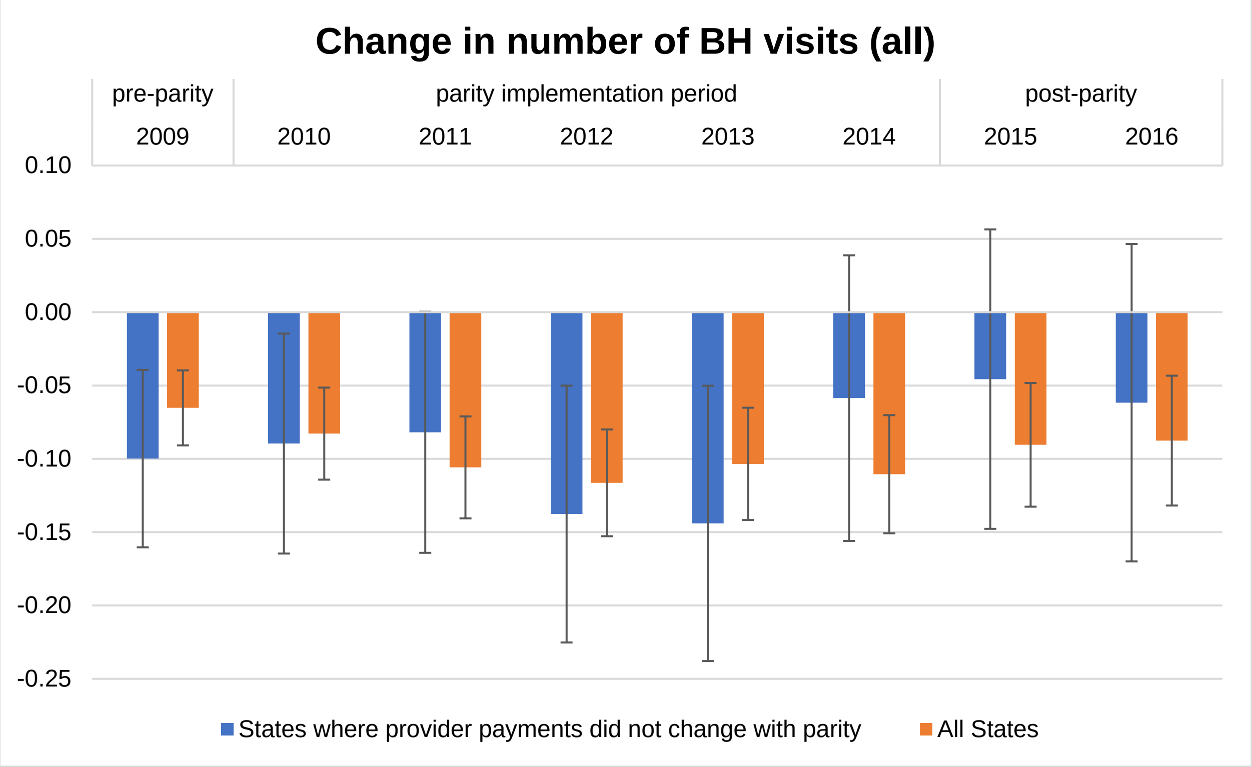

[a] We classified states without payment changes for duals as those with high Medicaid payment rates for all services (above 90% of the Medicare rate) or with full reimbursement policies for dual-eligibles (vs. lesser-of policies): AK, AR, DE, HI, IA, ME, MS, MO, MT, NE, ND, OH, OK, SD,TN, VT, WY.

© 2020 Fung V et al. *JAMA Network Open.*

Online-Only References

1. Kaiser Family Foundation. Medicaid-to-Medicare Fee Index. https://www.kff.org/medicaid/state-indicator/medicaid-to-medicare-fee-index/?currentTimeframe=0&sortModel=%7B%22colId%22:%22Location%22,%22sort%22:%22asc%22%7D. Published 2016. Accessed March 29, 2020.

© 2020 Fung V et al. *JAMA Network Open.*
